# Room-Temperature, Strong Emission of Momentum-Forbidden Interlayer Excitons in Nanocavity-Coupled Twisted van der Waals Heterostructures

**DOI:** 10.1021/acs.nanolett.4c05647

**Published:** 2025-01-08

**Authors:** Bin Feng, Shixuan Zhao, Ilya Razdolski, Feihong Liu, Zhiwei Peng, Yaorong Wang, Zhedong Zhang, Zhenhua Ni, Jianbin Xu, Dangyuan Lei

**Affiliations:** † Department of Materials Science and Engineering, Centre for Functional Photonics, and Hong Kong Branch of National Precious Metals Material Engineering Research Centre, City University of Hong Kong, Hong Kong S.A.R., 999077, China; ‡ Department of Physics, City University of Hong Kong, Hong Kong S.A.R., 999077, China; § Shenzhen Research Institute, City University of Hong Kong, Shenzhen, Guangdong 518057, China; ∥ School of Physics and Key Laboratory of MEMS of the Ministry of Education, Southeast University, Nanjing 211189, China; ⊥ Department of Electronic Engineering, The Chinese University of Hong Kong, Shatin Hong Kong S.A.R., 999077, China

**Keywords:** interlayer exciton, heterostructure, twist angle, Purcell effect, momentum mismatch

## Abstract

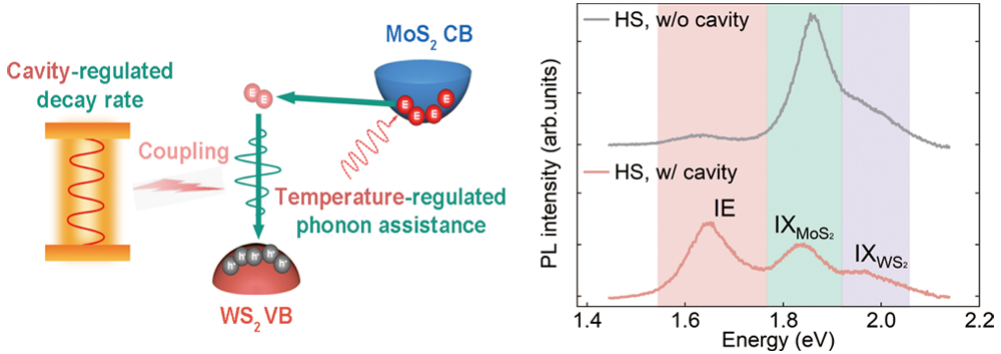

The emission efficiency of interlayer excitons (IEs) in twisted 2D heterostructures has long suffered from momentum mismatch, limiting their applications in ultracompact excitonic devices. Here, we report strong room-temperature emission of the momentum-forbidden IEs in a 30°-twisted MoS_2_/WS_2_ heterobilayer. Utilizing the Purcell effect of a compact plasmonic nanocavity boosts the IE emission intensity in the cavity by over 2 orders of magnitude. We further study the interplay of this Purcell enhancement and phonon assistance in 30°- and 0°-twisted heterostructures. Temperature-dependent and time-resolved spectroscopic measurements reveal that the IE enhancement in the 30°-twisted case involves competition between IE and intralayer-exciton emissions, which is remarkably distinct from the 0°-twisted case. We propose an exciton decay model capturing the features of phonon-assisted momentum compensation and Purcell enhancement in the IE emission, showing consistency with the experimental measurements. Our results enrich the understanding of the nanocavity-assisted light–matter interaction for momentum-indirect excitonic transitions.

Van der Waals heterostructures have emerged as a pioneering frontier in condensed matter physics and nanotechnology. Of particular interests are the heterostructures made from the transition metal dichalcogenides (TMD), offering a playground to explore novel quantum phenomena and device applications.^1,2^ An intriguing aspect of TMD physics arises from the type-II band alignment, which facilitates the spatial separation of electron–hole pairs. This leads to the formation of interlayer excitons (IE) with distinctive properties such as extended lifetimes, spatially indirect transitions, and permanent electrical dipole moments.^3,4^

Inspired by the unconventional superconductivity and Mott insulating states discovered in magic-angle twisted graphene,^5,6^ the interest in twisted TMD heterostructures has recently surged. By modulating the twist angle, the emission properties of TMD heterostructures can be readily engineered,^1,7^ which is promising for the development of ultracompact, on-chip, single-photon emitters and chiral light sources. However, it is well-known that the IE emission efficiency quenches markedly with the stacking angle arising from the momentum mismatch.^8−11^ So far, the IE emission is normally restricted to the heterostructures with close rotational alignment (stacking angle <10°).^1,3,12,13^ Particularly, when the twist angle approaches the maximum (30°), the remarkable mismatch in k-space between the valleys in the constituent monolayers leads to a superweak oscillator strength of the IE, typically for the momentum-forbidden transitions.^14−16^ The boost of forbidden excitonic emission has recently aroused great attention. Despite considerable efforts to enhance the emission of spin-forbidden excitons,^17−20^ similar advances in momentum-forbidden IE have yet to be witnessed.

Recently, the coupling of TMD heterostructures to nanocavities has proven effective in increasing the radiation efficiency of momentum-direct excitons through the Purcell effect.^17,21−24^ However, momentum-indirect IE transitions additionally require momentum-conserving phonon assistance^25,26^ and experience intricate competition with other relaxation processes such as intralayer exciton emission.^3^ So far, the detailed workings of the Purcell effect in these complex systems remain poorly understood, as momentum-indirect emission is not only regulated by the photon density of states but also requires exciton momentum compensation.

Here we achieve strong room-temperature emission of momentum-forbidden IE in 30°-twisted WS_2_/MoS_2_ heterostructures. By integrating the twisted heterostructure within a Au nanocube-on-mirror (NCoM) plasmonic nanocavity, we show that the Purcell effect can boost the IE emission efficiency, resulting in a photoluminescence enhancement factor of more than 2 orders of magnitude. We propose exciton decay models to investigate the impact of the phonon–exciton interaction and its interplay with the Purcell effect on the momentum-indirect IE emission.

Typically, IE emission can be divided into three steps:^3^ intralayer optical absorption, interlayer carrier transfer, and carrier recombination (Figure S1a). A large twist angle of the heterostructure induces a momentum mismatch between the adjacent valleys of the constituent monolayers (Figure S1b), which hinders carrier recombination and drastically reduces radiation efficiency.^14−16^ Note that the initial two stages are largely insensitive to the twist angle,^27−29^ indicating that sufficient carrier excitation and interlayer charge transfer can be ensured under arbitrary twist angles. Therefore, the bottleneck of the IE radiation efficiency lies solely in the final twist-angle-limited recombination stage, which may be overcome by the Purcell effect (Figure 1a). However, different from the Purcell-induced enhancement of the momentum-direct emission, the situation becomes more complex in the case of momentum-indirect transitions, where the Purcell enhancement may be influenced by additional requirements of momentum compensation. Due to the 3-fold rotation symmetry in k-space of the constituent TMD monolayers, 0° and 30° twist angle corresponds to the smallest and largest momentum mismatch, respectively.^29^ Therefore, we focus on the excitonic emission of heterostructures with these two twist angles in this work. Figure 1c shows a 30°-twisted WS_2_/MoS_2_ heterostructure in a NCoM plasmonic nanocavity, featuring a 100 nm Au Cube atop an ultrasmooth Au film (more fabrication details in Section S1). The WS_2_/MoS_2_ heterobilayer was inserted between the Au nanocube and the Au film, with a ∼3 nm thick hBN layer on the Au film to mitigate PL quenching.^23,30^ Polarization-resolved second-harmonic-generation (SHG) measurements confirmed a ∼30° stacking angle of the heterostructure (Figure 1d).

**Figure 1:**
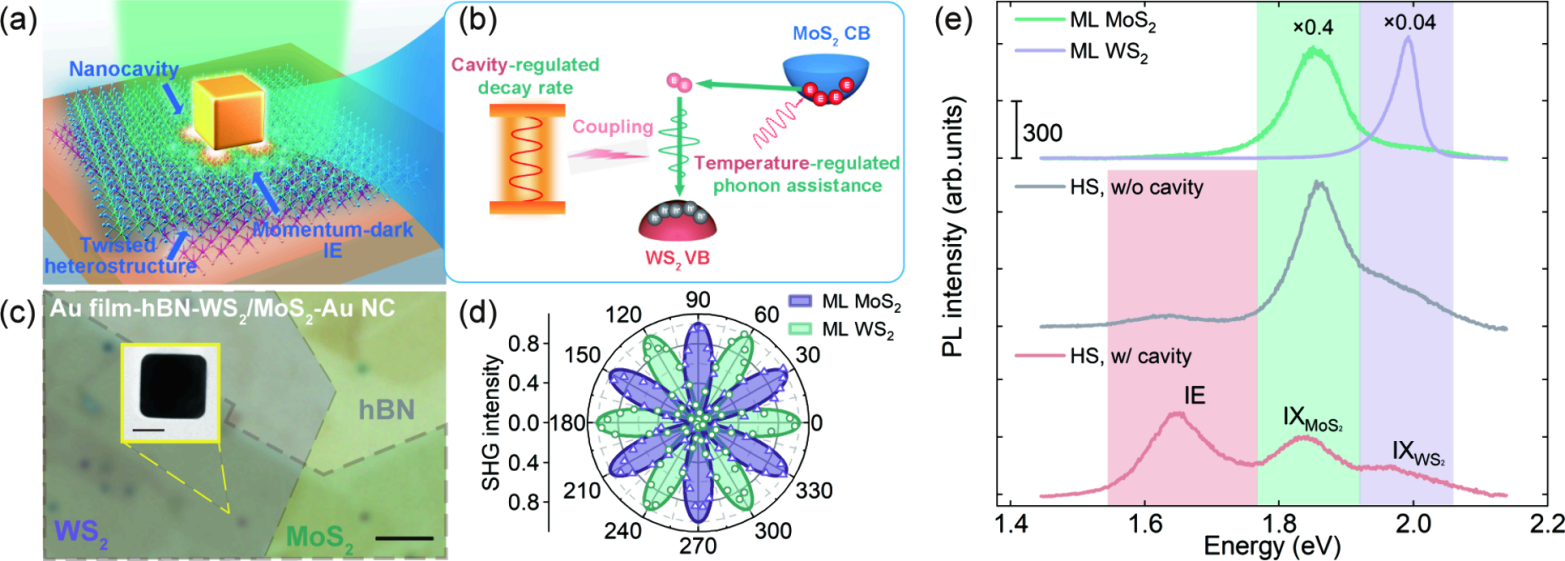
(a, b) Our strategy of combining phonon-assisted momentum compensation and nanocavity-induced Purcell effect to boost the momentum-indirect IE emission in twisted TMD heterostructures. (c) Optical image of a nanocavity-coupled 30°–twisted WS_2_/MoS_2_ heterostructure. Scale bar: 2 μm. Inset: TEM micrograph of a gold nanocube. Scale bar: 50 nm. (d) Polarization-resolved SHG measurements reveal a ∼30° stacking angle of the heterostructure in (c). The hollow points and solid curves are the measured and fitted polarization-dependent SHG intensity data, respectively, from individual monolayers (ML). (e) PL spectra from the nanocavity-coupled/uncoupled heterostructure (HS) and the surrounding monolayer regions.

As observed in multiple samples (Figure S2), the room-temperature PL performance of the ∼30°-twisted heterostructure coupled and uncoupled to the nanocavity shows a remarkable contrast. A representative case is shown in Figure 1e. In the uncoupled case, a spectral PL peak (1.86 eV) and a weak shoulder (1.99 eV) are indicative of the emission of the A-excitonic states in MoS_2_ and WS_2_ monolayers, respectively.^31,32^ Compared to the emission from the respective monolayers (upper panel, Figure 1e), the intralayer exciton (IX) emission in the heterostructure is notably quenched, indicating a good interlayer coupling.^3^ Besides the IX emission, no significant PL peaks are observed at lower energies, underscoring the intrinsically low IE radiation efficiency in the twisted heterostructures. Upon a closer inspection, a weak, broad peak is visible around 1.56–1.68 eV (Figure S3), consistent with the reported IE spectral range for WS_2_/MoS_2_ heterostructures.^3,22^ Notably, when the heterostructure is coupled to the nanocavity, the IE emission is significantly enhanced, even surpassing the emission intensity of IX. The nanocavity-enhanced emission is also confirmed by the PL mapping in the IE spectral range (1.56–1.68 eV), showing a bright spot at the nanocavity location (Figure S4). Considering that the emission enhancement occurs solely in the region covered by the nanocavity that is significantly smaller than the laser spot area, the PL enhancement factor is defined as *F* = (*I*_w/_*S*_tot_)/(*I*_w/o_*S*_cav_),^33,34^ where *I*_w/_ and *I*_w/o_ are the PL intensity of IE when coupled and uncoupled to the nanocavity, respectively. *S*_tot_ and *S*_cav_ are the areas of the laser spot and the nanocavity, respectively. In our case, the nanocavity coupling results in a PL enhancement factor of 188, which is comparable with the numbers reported previously for plasmonic cavity-enhanced excitonic emission.^22,33,35^ These findings clearly demonstrate a strong IE emission in twisted heterostructures induced by the nanocavity.

A careful design of the nanocavity geometry (nanocube size and gap distance) allowed us to achieve good spectral overlap of the cavity mode with the exciton emission line, which is key for the cavity-enhanced IE emission. The single-particle dark-field scattering on the heterostructure-coupled NCoM cavity (Figure 2a) shows two peaks at 1.65 and 2.34 eV. Numerical simulations (Figure S6) allowed to attribute them to the magnetic dipole (MD) and transverse (T) modes, respectively. In contrast, when the Au film is replaced by the SiO_2_ film, the MD mode vanishes, and no IE emission enhancement is observed (Figures S5 and S6). These findings indicate the indispensable role of the MD mode in the IE emission enhancement. The simulated surface charge distribution at the MD resonance (Figure 2b) shows opposite polarities at the bottom of the nanocube with mirror charges in the underlying Au film, thus creating a current loop which generates a localized magnetic field in the nanogap (Figure 2c). As a result, normal component of the electric field exhibits strong (∼10^2^) enhancement in the gap (Figure S7), which facilitates the radiative recombination of the IE with permanent out-of-plane dipole moment. As such, an effective excitonic emitter-cavity coupling is achieved, enabling the emission enhancement through the Purcell effect where the nanocavity boosts the local density of states and thus the transition rate.^33^

**Figure 2:**
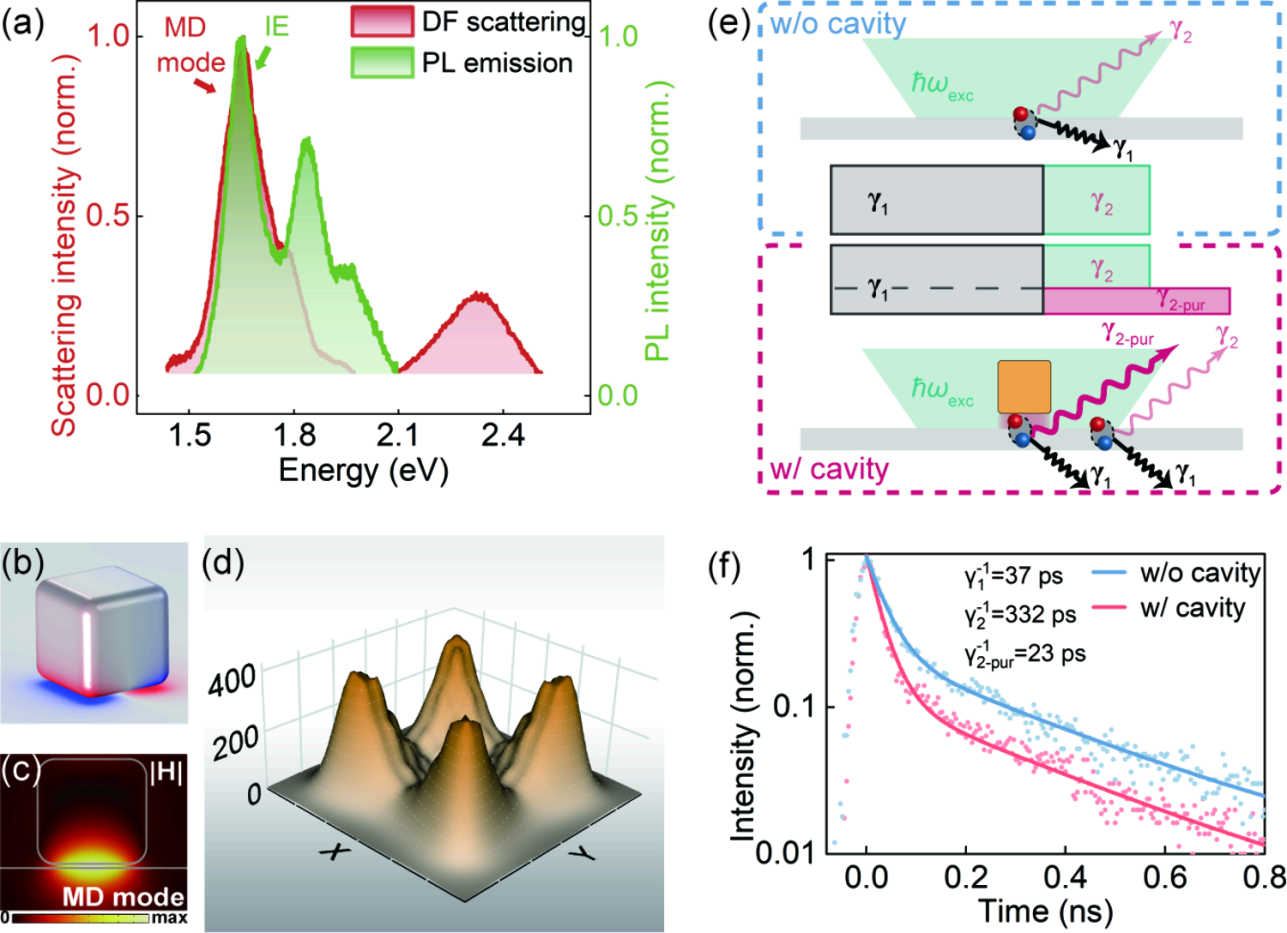
(a) Measured scattering and PL spectra for a heterostructure-coupled NCoM cavity. (b, c) Simulated surface charge and magnetic field distribution profiles at the MD resonance wavelength of the NCoM cavity in (a). (d) Simulated distribution of the radiative decay rate enhancement factor in the NCoM cavity. (e) Illustration of the experimental PL collection from a heterostructure without (top) and with a cavity (bottom). Various IE decay channels are shown with arrows. The green shaded area indicates the laser beam at ℏω_exc_ = 2.3 eV employed for the excitation. The inset in the middle schematically compares the decay rates diagram in the two cases. (f) TRPL spectra of IEs from a heterostructure without (light-blue) and with (purple) coupling to a NCoM cavity. The solid lines are the results of multi-exponential fitting of the experimental data.

This nanocavity-accelerated IE decay process was experimentally confirmed (Figure 2f) by systematic time-resolved PL (TRPL) analysis. For the case of a heterostructure without a nanocavity, a biexponential fitting of the IE response allowed identification of a fast (γ_1_^–1^ = 37 ps) and a slow (γ_2_^–1^ = 332 ps) decay component. The fast and slow processes were generally attributed to be dominated by the nonradiative and radiative decay, respectively.^36,37^ For the heterostructure coupled to a nanocavity, given that the laser spot diameter exceeds the nanocavity size, the situation becomes more complex due to the concurrent radiative and nonradiative decays, as well as the spatial inhomogeneity (i.e., collected signals contain contributions from both in-cavity and off-cavity regions, as shown in Figure 2e). From the TRPL measurement in Figure 2f, it can be observed that incorporating a nanocavity generally leaves the slow decay process unchanged while noticeably accelerating the fast part of the decay curve. Considering the cavity-enhanced PL intensity, the acceleration of the fast part should be mainly induced by the increase in the radiative decay rate. Therefore, when coupled to the nanocavity, the slow part of the decay curve is attributed to the radiative decay collected from the off-cavity region (γ_2_), which retains the properties of the heterostructure without a nanocavity. The fast part, however, can be further decomposed into an unchanged nonradiative process (γ_1_), and a strongly accelerated radiative decay process from the in-cavity region (γ_2-pur_).^34,38^ By simultaneously fitting the two decay curves based on the above understandings (Section S2), an additional decay component with 23 ps lifetime was extracted and attributed to the nanocavity-accelerated radiative decay process (γ_2-pur_). The Purcell factor, i.e., the ratio of the radiative decay rates with and without the cavity, was estimated to be 14.4 (γ_2_^–1^/γ_2-pur_^–1^). We further performed numerical simulations to obtain the mapping of the enhancement of the radiative decay rate (Figure 2d) for the dipole emitters oriented normally to the surface in resonance with the MD mode. The calculated decay rate enhancement varies spatially within the nanogap, peaking at ∼400 at the nanocavity corners. Additionally, the near-field excitation enhancement alone shows negligible contribution to the IE emission enhancement (Figures S5 and S6). These findings confirm the Purcell-driven boost of momentum-indirect IE emission in the nanocavity.

Momentum-indirect IE emission in twisted heterostructures requires phonon-assisted momentum compensation and competes with other processes such as IX emission, which is regulated by temperature. It is reported that new emission features (such as the emission from localized states and the excitons bound to surface adsorbates) will emerge^39^ and moire effect becomes significant^40^ at low temperature, which will complicate the analysis at this stage. Therefore, in the following we investigate the temperature-regulated excitonic properties under elevated temperature from 293–393 K. As shown in Figures S8 and S9, there is no significant change in the morphology or the scattering spectrum of the Au cubes after heating. Figure 3a displays the temperature-dependent PL spectra from a cavity-coupled heterostructure. Deconvolution of the observed PL response into Gaussian components allowed us to analyze the temperature dependence of the three excitonic contributions (Figure 3b). First, it is seen that the relative contribution of WS_2_ IX is small, presumably due to the interlayer coupling-induced PL quenching, and exhibits weak variations. The MoS_2_ exciton emission intensity increases with temperature. This phenomenon was also observed in monolayer MoS_2_ (Figure S10) and reported previously, which was ascribed to the enhanced trion-to-exciton conversion at higher temperature.^41−43^ As for the IE emission, its intensity shows a nonmonotonic behavior, indicating a competition of multiple contributing factors. In what follows, we focus on the intensity ratio of the IE and MoS_2_ IX contributions, which reveals the competition between the IE and IX dynamics. The ratio *I*_IE_/*I*_MoS_2__ was calculated by using the integrated intensity of each deconvolved peak. We start from the cases of cavity-uncoupled heterostructures. Interestingly, the *I*_IE_/*I*_MoS_2__ ratio in the 0°-twisted case continuously decreases with temperature, which is distinct from the nonmonotonic behavior observed in the 30°-twisted case (Figure 3c). Note that in all cases, *I*_MoS_2__ increased with temperature (Figures S11 and S12). Thus, the continuous decrease of *I*_IE_/*I*_MoS_2__ in the 0°–twisted case indicates that IE emission becomes less competitive at elevated temperatures. Nevertheless, for the 30°–twisted case, there is a clear rise of the *I*_IE_/*I*_MoS_2__ ratio during the initial temperature increase (293–333 K), indicating a stronger IE emission capability at higher temperature.

**Figure 3:**
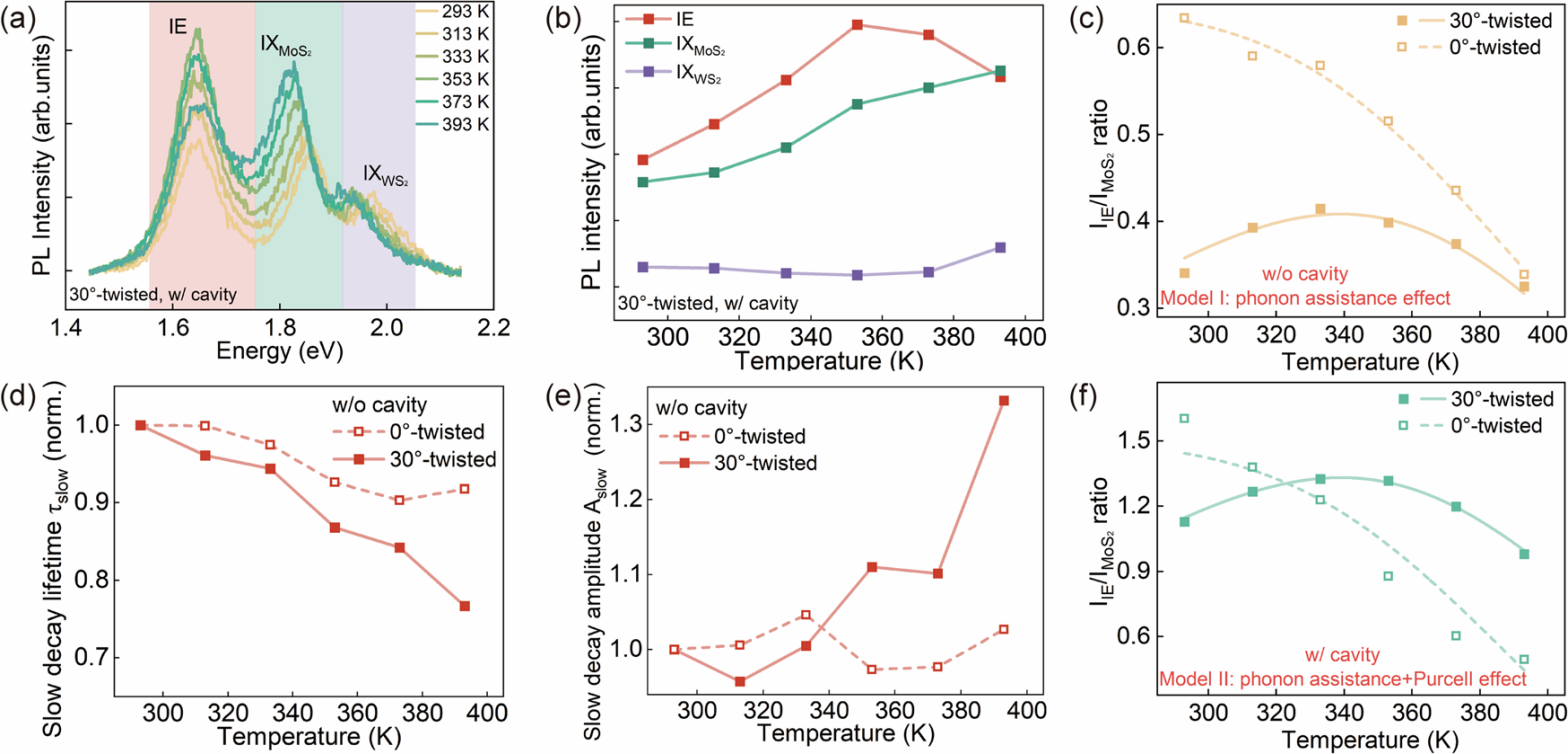
(a, b) Temperature-dependent PL spectra and the deconvolved emission intensity of the three exciton states in a nanocavity-coupled 30°-twisted heterostructure. (c) Temperature-dependent *I*_IE_/*I*_MoS_2__ ratio obtained on the pristine 0°/30°-twisted heterostructures. (d, e) Variations of the slow decay lifetime and its amplitude with temperature in the pristine 0°/30°-twisted heterostructures. For better comparison, the values are normalized to those at room temperature. (f) Temperature-dependent *I*_IE_/*I*_MoS_2__ ratio obtained on the nanocavity-coupled 0°/30°-twisted heterostructures. The data points and curves in (c) and (f) are the experimental results and corresponding fitting based on the decay models, respectively.

The contrasting trends of *I*_IE_/*I*_MoS_2__ ratio between the 0°- and 30°-twisted heterostructures indicate the important role of phonon-assisted momentum compensation, which is a thermally enhanced process. Similar conclusions can be reached when analyzing the temperature-dependent TRPL response. As mentioned above, the radiative decay governs the slow component of the TRPL response in the cavity-uncoupled heterostructure. As the temperature increases, we observed a more pronounced acceleration of the radiative decay in the 30°-twisted case than that in the 0°-twisted case (Figure 3d). This radiative contribution can be characterized by its weight *A*_slow_ in the fitting function . The 30°-twisted heterostructures exhibit an increase in *A*_slow_ with temperature, which is not observed in the 0°-twisted case (Figure 3e). These discrepancies further confirm a stronger effect of the temperature on the IE emission efficiency in the 30°-twisted heterostructures where the momentum-indirect IE recombination requires phonon assistance.

To understand the PL response of the cavity-uncoupled 0°/30°-twisted heterostructures, we propose an exciton decay model capturing the temperature-dependent emission features by considering the phonon–exciton interaction (Model I, Section S3). The phonon-assisted IE emission intensity reads: *I*_IE_ ∝ *N*_trans_*f*_phonon_*Q*_IE_, where *N*_trans_ is the amount of interlayer-transferred carriers, *Q*_IE_ is the quantum yield of IE, *f*_phonon_ = 1 + 2*n*_phonon_ represents the effect of the phonon-assisted momentum compensation accounting for both phonon generation (∝1 + *n*_phonon_) and consumption (∝*n*_phonon_), *n*_phonon_ = (*e*^*E*_p_/*k*_B_*T*^ – 1)^−1^ is the population of thermal phonons with an energy *E*_p_ based on Bose–Einstein statistics, and *k*_B_ is the Boltzmann constant. In turn, the intensity of MoS_2_ IX comprises contributions from both excitons and trions: *I*_MoS_2__ ∝ *N*_remain_[β*Q*_neu_ + (1 – β)*Q*_tri_]. Here, *N*_remain_ is the amount of carriers remaining in the original layer without interlayer transfer. *Q*_neu_ and *Q*_tri_ are the quantum yields of the MoS_2_ excitons and trions, respectively, and β is the proportion of the MoS_2_ excitons. Analyzing the exciton-trion equilibration, the temperature dependence of β can be described by *ae*^–*E*_b_/*k*_B_*T*^/(1 + *ae*^–*E*_b_/*k*_B_*T*^), where *E*_b_ is the energy difference between the final state (trions) and the initial state (excitons and electrons) and *a* is a parameter involving Arrhenius factor and the electron density. Finally, the *I*_IE_/*I*_MoS_2__ can be derived as follows:
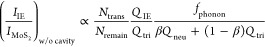
1

Equation 1 describes the competition between the emission of MoS_2_ IX and phonon-assisted momentum-indirect IE in 30°-twisted heterostructures without a nanocavity. Normally, excitons exhibit higher radiation efficiency than trions, and the trion-to-exciton conversion is enhanced at higher temperatures.^41−43^ The competition between the growing *f*_phonon_ and the increasing overall quantum yield of the MoS_2_ exciton-trion ensemble results in a nonmonotonic behavior of *I*_IE_/*I*_MoS_2__ with temperature (Figure 3c). In contrast, in the 0°-twisted case, the IE recombination is momentum-direct and requires no phonon assistance. Without the *f*_phonon_ term, the *I*_IE_/*I*_MoS_2__ ratio continuously decreases with the temperature. As shown in Figure 3c, decay Model I reproduces the experimental results well for both the 0°- and 30°-twisted heterostructures.

We further investigated cavity-coupled heterostructures. Notably, the contrast in the temperature-dependent evolution of the *I*_IE_/*I*_MoS_2__ ratio between 0°- and 30°-twisted samples was also observed (Figure 3f), similar to the cavity-uncoupled cases. This indicates that the phonon-assisted effect also influences the heterostructure-cavity coupled system. We extended the decay Model I by simultaneously considering phonon–exciton interaction and the Purcell effect (see more details of the Model II in Section S4). The Purcell effect contributes to an enhancement of *Q*_IE_ that can be quantified by Purcell factor *P*_f_. We note that in our experiment, the PL signal is obtained from the whole laser-irradiated region (*S*_tot_), which is much larger than the nanocavity region (*S*_cav_) producing the Purcell effect. Therefore, the influence of the Purcell effect on *I*_IE_/*I*_MoS_2__ should be calibrated by considering the size discrepancy between the laser spot and the nanocavity. Based on these analyses, when the heterostructure is coupled with the NCoM nanocavity, *I*_IE_/*I*_MoS_2__ can be described as follows:
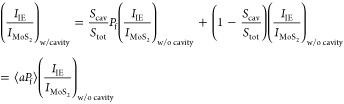
2where  is the apparent Purcell factor to describe the overall enhancement across the whole area of the laser spot. As shown in Figure 3f, the excellent fit quality corroborates the validity of the extended model (Model II) describing the phonon- and cavity-assisted IE emission. The apparent Purcell factor ⟨*aP*_f_⟩ derived within Model II is 3.98, which is close to the value obtained from the TRPL experiment (Section S5). Based on these understandings, we elucidated the mechanism for momentum-indirect IE transitions in the cavity-coupled heterostructure (Figure 1b): The interlayer-transferred carriers initially form interlayer excitons, which then interact with phonons to acquire the necessary momentum for the momentum-direct while spatially indirect radiative decay process. This thermally enhanced, phonon-assisted momentum compensation is supported by temperature-dependent spectroscopic experiments and decay model analysis. When further integrated with the nanocavity, the radiative decay process is modulated through the Purcell effect by cavity-heterostructure coupling. Therefore, both the phonon–exciton interaction and the Purcell effect contribute to the momentum-indirect IE emission, which can be regulated by temperature and cavity design, respectively.

Here we have an outlook for future research. In this work, we investigated two most representative cases (0° and 30° twist angle) that correspond to the situation of the smallest and largest momentum mismatch. For the case of intermediate twist angles, the aforementioned factor describing the phonon-assisted momentum compensation can be further expressed as , where θ is the twist angle. According to the acoustic phonon dispersion of TMD monolayers,^44,45^ a larger twist angle leads to a higher *E*_p_ of thermal phonons for momentum compensation. Therefore, compared with the case of the 30° twist angle, intermediate twist angles will have a lower *E*_p_ and correspondingly a larger *f*_phon_, which indicates an easier momentum compensation for the interlayer exciton emission. Since the twist angle will not substantially shift the broad peak of interlayer excitons, the overlap of the cavity mode with the exciton emission peak is still guaranteed. Besides, the magnitude of the Purcell effect (i.e., Purcell factor) is proportional to the wavelength, quality factor, and mode volume of the cavity mode.^17^ These properties are generally insensitive to the twist angles of the heterostructures. Therefore, the Purcell effect is preserved for the case of intermediate angles. The above physical picture of twist angle-regulated momentum mismatch is widely adopted by previous reports.^3,13,46,47^ Besides, there may be broader roles played by the twist angle. On the one hand, a larger twist angle may increase the interlayer distance of the heterostructure,^9^ which affects the interlayer exciton emission. On the other hand, twist angle can also induce the moire effect that induces spatially localized, periodic potential wells in heterostructures, which introduces additional features for exciton emission at low temperature.^1^ Finally, regarding sample fabrication, the emission of interlayer excitons depends on the intimate contact between the two TMD monolayers, which is influenced by various practical factors. Future efforts could aim to enhance heterostructure uniformity by using monolayers with consistent properties, minimizing strain and bubbles during stacking and achieving better control of twist angles.

In summary, we demonstrated strong room-temperature emission of momentum-forbidden IE in a 30°-twisted WS_2_/MoS_2_ heterobilayer. By integrating the heterostructure with an NCoM plasmonic nanocavity, the momentum-indirect excitonic emission intensity in the cavity was boosted by more than 2 orders of magnitude through the Purcell effect. Time-resolved and temperature-dependent PL reveals markedly distinct competition between IE and intralayer−exciton emissions in 30°- and 0°-twisted structures. To understand the phonon-assisted momentum compensation interplaying with the Purcell effect, we developed an exciton decay model that shows good agreement with the experimental measurements. The strongly enhanced emission of momentum-forbidden IE will facilitate novel optoelectronic applications based on twisted heterostructures. Besides, these results reveal that temperature-regulated phonon–exciton interaction and cavity-induced Purcell effect are both crucial for the effective momentum-indirect transition.

## Supporting Information Available

Details of sample fabrication and characterization method, detailed lifetime analysis based on TRPL experiments, the elucidation of decay Models I and II, and experiment data fitting based on the proposed decay models. Supporting figures, including schematic illustration of the IE formation steps, supplementary PL characterization, cavity mode analysis, the heating effect on the nanocavities, temperature-dependent PL investigation, and the instrument response function of our detection system (PDF)
