# Supplementary material for: Room-Temperature, Strong Emission of Momentum-Forbidden Interlayer Excitons in Nanocavity-Coupled Twisted van der Waals Heterostructures
Source: Nano Lett. 2025 Jan 8;25(4):1609–16. doi: 10.1021/acs.nanolett.4c05647 (PMC11783585; doi:10.1021/acs.nanolett.4c05647)
Supplement: Supplementary file 1 — nl4c05647_si_001.pdf [file nl4c05647_si_001.pdf]

Room-Temperature, Strong Emission of Momentum-Forbidden Interlayer Excitons in Nanocavity-Coupled Twisted Van der Waals Heterostructures

Bin Feng[1], Shixuan Zhao[1,2], Ilya Razdolski[1], Feihong Liu[1], Zhiwei Peng[1], Yaorong Wang[1], Zhedong Zhang[2,3], Zhenhua Ni[4], Jianbin Xu[5], Dangyuan Lei[1,2*]

[1] Department of Materials Science and Engineering, Centre for Functional Photonics, and Hong Kong Branch of National Precious Metals Material Engineering Research Centre, City University of Hong Kong, Hong Kong S.A.R., 999077, China.
[2] Department of Physics, City University of Hong Kong, Hong Kong S.A.R., 999077, China.
[3] Shenzhen Research Institute, City University of Hong Kong, Shenzhen, Guangdong 518057, China.
[4] School of Physics and Key Laboratory of MEMS of the Ministry of Education, Southeast University, Nanjing 211189, China.
[5] Department of Electronic Engineering, The Chinese University of Hong Kong, Shatin 999077, Hong Kong SAR, China
* E-mail: dangylei@cityu.edu.hk

Sec. 1 Materials and Methods

Heterostructure fabrication and nanocavity integration

The heterostructure was fabricated by a standard dry transfer technique. ~3 nm thick hBN flake was mechanically exfoliated from the single bulk crystal using a scotch tape, and then transferred onto a ~200 nm-thick, template-stripped gold mirror[1, 2] through a polydimethylsiloxane film for viscoelastic stamping. Then the MoS_2 and WS_2 monolayers were also mechanically exfoliated and transferred onto the hBN flakes. The thickness of the MoS_2 and WS_2 layers was initially identified with the help of optical microscopy, and further examined by steady-state PL and Raman measurements[3, 4]. The thickness of the hBN flakes was determined by the AFM measurements. Subsequent to the materials assembly, the heterostructure was annealed in a furnace at 200 °C for 6 h in a high vacuum (10^{-4} Pa). 100 nm-large Au nanocubes coated with ~2 nm-thick cetyltrimethylammonium bromide (CTAB) layer were dispersed onto the heterostructures to form an integrated architecture comprising a hBN flake and a MoS_2 /WS_2 bilayer inside the Au cube-on-mirror nanocavity. The hBN flake and CTAB coating of nanocubes mitigate the charge transfer between the metal and the heterostructure[1, 5]. The twist angle of the WS_2/MoS_2 heterobilayer was initially realized during the transfer process based on the cleavage features along the preferred crystallographic orientation of the 2D TMD materials[6]. After the fabrication, the twist angle of the heterobilayer was further confirmed by polarization-resolved SHG measurements [7].

Optical spectroscopy

The steady-state PL measurements were performed with the help of a commercial Confocal Raman Microscope (WITec Alpha300R). A 532-nm continuous wave laser was introduced to the confocal system and focused on the samples using a 100× objective (NA=0.9). The spot diameter (distance from center where intensity drops to $1/e^2$) was determined by the diffraction-limited regime[8, 9]: $d=0.82\lambda/NA$, where NA and λ are the numerical aperture of the objective and the excitation laser wavelength,

respectively. The temperature-dependent measurements were carried out by further integrating a heating stage (INSTEC stage) under the objective. The time-resolved PL (TRPL) measurements were carried out based on a time-correlated single photon counting module. A mode-locked Ti: Sapphire laser-pumped OPA with a pulse duration of ~150 fs, repetition rate of 80 MHz, and wavelength of 532 nm was employed. The TRPL response was collected with an avalanche photodiode, whereas narrowband spectral filters were placed in the detection path to selectively cut the IE emission spectrum. Optical images and dark field scattering spectra were obtained using an optical microscope (Olympus DP72, 100× objective, NA=0.8).

SHG measurements were performed in a home-built confocal microscope setup. The 800 nm output of a Ti:sapphire laser oscillator (pulse width 150 fs, central wavelength 800 nm) was coupled into the 100× microscope objective. A linear polarizer was inserted into both the excitation and detection paths, so that SHG radiation polarized parallel to the excitation polarization was detected. Short-pass filters were employed to separate the SHG radiation from the reflected fundamental laser light. The collected SHG signal was coupled into a grating spectrometer (Andor SR500) and detected with a Peltier-cooled charge-coupled device.

Numerical Simulations

Full-wave electromagnetic simulations for the plasmonic mode analysis were conducted using COMSOL Multiphysics V6.1, which utilized the finite element method. The permittivity of gold was taken from the empirical data given by Johnson and Christy[10]. The entire computational domain was enclosed by a perfectly matched layer to minimize unwanted reflections at the domain boundaries. The scattering spectra of the nanocavity were derived by integrating the upward scattering power flow within a solid angle of 103 degrees, which corresponds to the numerical aperture (NA) of 0.8 of the objective lens used in the experiments. The meshing of the simulation models, particularly in the gap region, was carefully refined to ensure computational convergence. For the simulation of the radiative decay rate enhancement of interlayer

excitons in an NCoM cavity, the MATLAB toolbox MNPBEM was used to solve the Maxwell's equations[11].

Sec. 2 lifetime analysis based on TRPL experiments

As elucidated in the main text regarding Fig. 2e, f, for the case of a heterostructure without a nanocavity, a fast (γ_1^{-1}) and a slow (γ_2^{-1}) decay component were attributed to be dominated by the nonradiative and radiative decay, respectively. For the heterostructure coupled to a nanocavity, given that the laser spot diameter exceeds the nanocavity size, the slow part of the decay curve is attributed to the radiative decay collected from the off-cavity region (γ_2), which retains the properties of the heterostructure without a nanocavity. The fast part, can be further decomposed into an unchanged nonradiative process (γ_1), and a strongly accelerated radiative decay process from the in-cavity region ($\gamma_{2-\mathrm{pur}}$).

We used the global fitting method to extract these decay components: The two decay curves in Fig. 2f were simultaneously fitted using a bi-exponential and a tri-exponential fitting function, respectively. Based on the elucidation in the main text, the two fitting functions share common γ_1 and γ_2 during fitting. Finally, the three decay components were then determined to be γ_1^{-1}=37 ps, γ_2^{-1}=332 ps, and $\gamma_{2-\mathrm{pur}}$=23 ps, respectively.

Sec. 3 Decay model I: the effect of phonon-assisted momentum compensation

To describe the distinct behavior of the temperature-dependent PL intensity ratio $I_{IE}/I_{\mathrm{MoS_2}}$ between the 30°– and 0°–twisted case, we proposed a decay model by considering the phonon-assisted momentum compensation for the indirect emission process, which is a thermal-enhanced process. Fig. S1a illustrates the formation of IE, which consists of three steps: 1: Intralayer optical absorption. 2: Interlayer carrier transfer to form IE. 3: Carrier recombination. In our experiment, the 30°– and 0°–twisted cases have the same material configuration (i.e., WS_2/MoS_2 heterostructure). The carrier excitation and interlayer carrier transfer are reported to be twist angle-independent[16-18].The excitation parameters are the same for both cases. Based on these conditions, the amount of interlayer-transferred carriers N_{trans}, and the amount of carriers remaining in the original layer without interlayer transfer $N_{\mathtt{remain}}$ can be regarded as constants for all the investigated cases.

For the 0°–twisted case, the PL intensity of interlayer excitons I_{IE} is proportional to the amount of interlayer-transferred carriers N_{trans}, and the quantum yield Q_{IE} (the ratio of the radiative decay rate to the total decay rate of IE).

For the 30°–twisted case, the emission of momentum-indirect IE involves the phonon assistance to compensate for the momentum mismatch. Therefore, we claim that only a small proportion of the carries that gain the required momentum from exciton-phonon interaction is capable of the subsequent radiative recombination (Fig. 1b). Then the PL intensity of interlayer excitons, I_{IE} is proportional to an extra term f_{phonon} describing the effect of the phonon-assisted momentum compensation.

As for the PL intensity of MoS_2 intralayer excitons $I_{\mathrm{MoS_2}}$, due to the type-II band alignment (Fig. S1a), only a small proportion of carriers (holes for MoS_2) remains within the MoS_2 layer in the heterostructure. The emission intensity $I_{\mathrm{MoS_2}}$ is limited by the amount of remaining carriers in the original layer without the interlayer transfer $N_{\mathtt{remain}}$. In the experiment, we observed an enhanced $I_{\mathrm{MoS_2}}$ at elevated temperature (Fig. 3b and S10). This phenomenon has also been reported previously, and attributed

to the trion-to-exciton conversion enhanced at elevated temperature due to the higher radiation efficiency of excitons than that of trions[12]. Therefore, $I_{\mathrm{MoS_2}}$ is proportion to N_{remain}, as well as the relative proportion of MoS_2 excitons and trions, and the corresponding quantum yield.

Based on the above analysis, the quantity $I_{IE}/I_{\mathrm{MoS_2}}$ can be described using the following formula:

$$\left(\frac{I_{IE}}{I_{\mathrm{MoS_2}}}\right)_{\mathrm{w/o\ cavity}} \propto \frac{N_{trans}}{N_{\mathrm{remain}}}\frac{Q_{IE}}{Q_{\mathrm{tri}}}\frac{f_{phonon}}{\beta Q_{neu}+(1-\beta)Q_{tri}}(S1)$$

where Q_{neu} and Q_{tri} are the quantum yield of MoS_2 intralayer excitons and trions, respectively.β is the proportion of the MoS_2 neutral excitons.

Equation S2 is further elaborated in the following. The effect of the phonon-assisted momentum compensation f_{phonon} can be understood as follows. For the radiative recombination of momentum-indirect interlayer excitons, the momentum mismatch k_{Δ} can be compensated by either emission or absorption of a phonon. Accounting for both spontaneous and stimulated scattering, the probabilities of these two processes are proportional to $1+n_{phonon}$ and n_{phonon}, respectively, so that, $f_{phonon}=1+2n_{phonon}$. In turn, n_{phonon} is the population of thermal phonons with energy E_p based on Bose-Einstein statistics:

$$n_{phonon}=\frac{1}{e^{\frac{E_p}{k_B T}}-1}(S2)$$

where T is the temperature and k_B is the Boltzmann constant.

The trion-to-exciton conversion process $Tr\rightarrow X+\mathrm{e}^{-}$ can be further deduced from the chemical equilibrium theory. When reaching the equilibrium, the conversion rate for the forward and the backward process should be equal:

$$\mathrm{k}_1\mathrm{n}_T=\mathrm{k}_{-1}\mathrm{n}_X\mathrm{n}_{\mathrm{e}^-}(S3)$$

where k_1 and k_{-1} are the rate constant of the forward and the backward process, respectively. n_X, n_T, and $\mathrm{n}_{\mathrm{e}^-}$ represent the density of the MoS_2 excitons, trions, and the electrons, respectively. Arrhenius equation describes the temperature dependence of reaction rates:

$$k = A_{arr} e^{\frac{-E_a}{k_B T}} (S4)$$

where T is the absolute temperature, A_{arr} is the Arrhenius factor, E_a is the activation energy for the conversion.

By transforming Equation S4, we can obtain:

$$\frac{\mathrm{n}_X}{\mathrm{n}_T} = \frac{k_1}{\mathrm{n_e}\text{–}k_{-1}} = \frac{A_1}{\mathrm{n_e}\text{–}A_{-1}} e^{\frac{-(E_1 - E_{-1})}{k_B T}} = a e^{\frac{-E_b}{k_B T}} (S5)$$

where E_b is the energy difference between the initial state (trions) and the final state (excitons and electrons). Then, the proportion of MoS_2 intralayer neutral excitons β is obtained:

$$\beta = \frac{\mathrm{n}_X}{\mathrm{n}_X + \mathrm{n}_T} = \frac{a e^{\frac{-E_b}{k_B T}}}{1 + a e^{\frac{-E_b}{k_B T}}} (S6)$$

The quantum yield is the ratio of the radiative decay rate to the total decay rate (radiative and nonradiative). As the quantum yield of TMD materials is normally very low (normally below 2%[13]), the variation of nonradiative components with temperature will not significantly change the quantum yield. Therefore, the quantum yield terms Q_{IE}, Q_{neu}, and Q_{tri}, can be regarded as constants. Combining all the above equations, for the 0°–twisted case with direct IE emission, $I_{IE}/I_{\mathrm{MoS_2}}$ can be described as follow:

$$\left(\frac{I_{IE}}{I_{\mathrm{MoS_2}}}\right)_{w/o\ \mathrm{cavity,\ direct\ emission}} \propto \frac{N_{trans}}{N_{\mathrm{remain}}} \frac{Q_{IE}}{Q_{\mathrm{tri}}} \frac{1}{\beta Q_{neu} + (1-\beta) Q_{tri}} \propto \frac{C}{(B-1)\frac{a e^{\frac{-E_b}{k_B T}}}{1 + a e^{\frac{-E_b}{k_B T}}} + 1} \quad (S7)$$

where the parameter C involves the ratio $N_{trans}/N_{\mathrm{remain}}$, and Q_{IE}/Q_{tri}. B is the ratio $Q_{\mathrm{neu}}/Q_{\mathrm{tri}}$.

For the 30°–twisted case with indirect IE emission, the effect of the phonon-assisted momentum compensation should be additionally considered, obtaining the following:

$$\left(\frac{I_{IE}}{I_{\mathrm{MoS_2}}}\right)_{w/o\ \mathrm{cavity,\ indirect\ emission}} \propto \frac{N_{trans}}{N_{\mathrm{remain}}} \frac{Q_{IE}}{Q_{\mathrm{tri}}} \frac{f_{phonon}}{\beta Q_{neu} + (1-\beta) Q_{tri}} = f_{phon} \left(\frac{I_{IE}}{I_{\mathrm{MoS_2}}}\right)_{w/o\ \mathrm{cavity,\ direct\ emission}} \quad (S8)$$

Sec. 4 Decay model II: the effects of phonon-assisted momentum compensation and the Purcell enhancement

The momentum-indirect IE transitions from the cavity-coupled heterostructure involve both phonon-assisted momentum compensation and the Purcell effect (Fig. 1b): The interlayer-transferred carriers initially form interlayer excitons, then interact with phonons to acquire the necessary momentum, followed by a momentum-direct while spatially-indirect radiative decay process that can be modulated through the Purcell effect.

The quantum yield of TMD materials is normally very low (normally below 2%[13]). Under the low quantum yield limit, the quantum yield enhancement can be approximated as the radiative decay rate enhancement[14]. Therefore, the Purcell effect contributes to an enhancement of Q_{IE} that can be quantified by the Purcell factor P_{f}. We note that in our experiment, the PL signal is obtained from the whole laser-irradiated region, which is much larger than the nanocavity region producing the Purcell effect. Therefore, the influence of the Purcell effect on $I_{IE}/I_{\mathrm{MoS_2}}$ should be calibrated considering the size discrepancy between the laser spot and the nanocavity. Based on these analyses, when the heterostructure is coupled with the NCoM nanocavity, $I_{IE}/I_{\mathrm{MoS_2}}$ can be described as follow:

$$\begin{aligned}\left(\frac{I_{IE}}{I_{\mathrm{MoS_2}}}\right)_{w/\,\mathrm{cavity}} &= \frac{S_{cav}}{S_{tot}}P_f\left(\frac{I_{IE}}{I_{\mathrm{MoS_2}}}\right)_{w/o\,\mathrm{cavity}} + \left(1-\frac{S_{cav}}{S_{tot}}\right)\left(\frac{I_{IE}}{I_{\mathrm{MoS_2}}}\right)_{w/o\,\mathrm{cavity}} \\ &= \left[(P_f-1)\frac{S_{cav}}{S_{tot}}+1\right]\left(\frac{I_{IE}}{I_{\mathrm{MoS_2}}}\right)_{w/o\,\mathrm{cavity}} \\ &= \langle aP_f\rangle\left(\frac{I_{IE}}{I_{\mathrm{MoS_2}}}\right)_{w/o\,\mathrm{cavity}}\end{aligned} \quad \text{(S9)}$$

where $\langle aP_f\rangle = (P_f-1)\frac{S_{cav}}{S_{tot}}+1$ is the apparent Purcell factor to describe the overall enhancement across the whole area of the laser spot. S_{cav} and S_{tot} are the area of the nanocavity region and the laser spot, respectively. As elucidated in the main text, the Purcell factor P_f was determined to be 14.4 from the measured lifetime in Fig. 2f. By substituting this P_f into Equation S1, $\langle aP_f\rangle$ was calculated to be 1.73.

Sec. 5 Experiment data fitting based on the proposed decay model I and II

The measured temperature-dependent behavior of $I_{IE}/I_{\mathrm{MoS_2}}$ in the four cases (i.e., 0°/30°–twisted heterostructures coupled/uncoupled with the nanocavity) shown in Fig. 3c, f can be fitted based on the above equations S7–9. In our fitting, the quantum yield ratio $Q_{\mathrm{neu}}/Q_{\mathrm{tri}}$ was set to be 6, as obtained from previous reports[15]. The energy of phonons E_{p} required for the momentum compensation was determined to be 0.013 eV according to the phonon dispersion of monolayer MoS_2. To improve the stability the proposed model, we simultaneously fitted the experimental data for all samples: i.e., the parameters C, E_{b}, and a were restricted to be identical. As shown in Fig. 3c, f, and Fig. S13, the fitting produced a good agreement with the experimental results.

The $\langle aP_f \rangle = 3.66$ was obtained from the model fitting, while the value obtained from lifetime experiments is 1.73 (more details in Sec. S4). Similar with many previous reports regarding nanocavity-enhanced fluorescence,[16-18] the experimentally obtained P_f represents only a lower bound due to the limited time resolution of the TRPL measurements (~21 ps, Fig. S14). Correspondingly, the value of 1.73 is the lower bound of the apparent Purcell factor $\langle aP_f \rangle$ corrected by the sizes of the laser spot and the nanocavity.

Supporting Figures

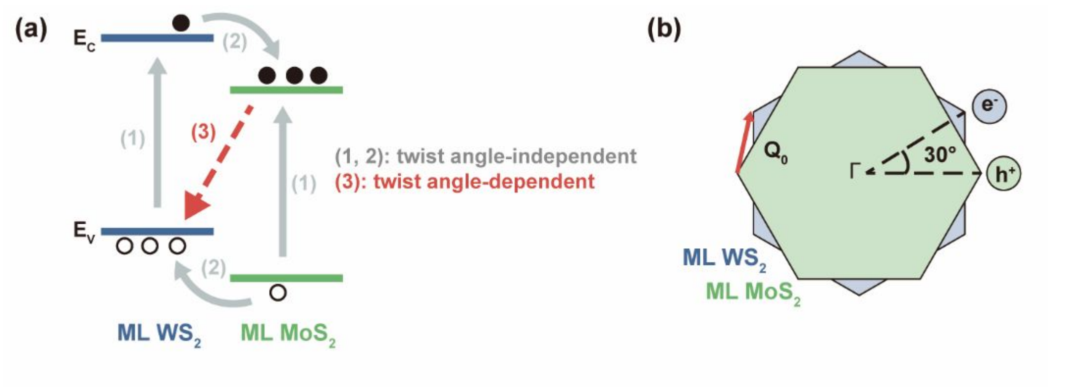

Figure S1. (a) The schematic illustration of the IE formation steps in WS_2/MoS_2 heterostructures: 1: Intralayer optical absorption. 2: Interlayer carrier transfer enabling the formation of IE. 3: Carrier recombination. While the third step is twist angle-dependent[19-22], the former two steps are twist angle-independent[23-25]. (b) The schematic illustration of the first Brillouin zone in monolayer MoS_2 and WS_2 twisted in the momentum space.

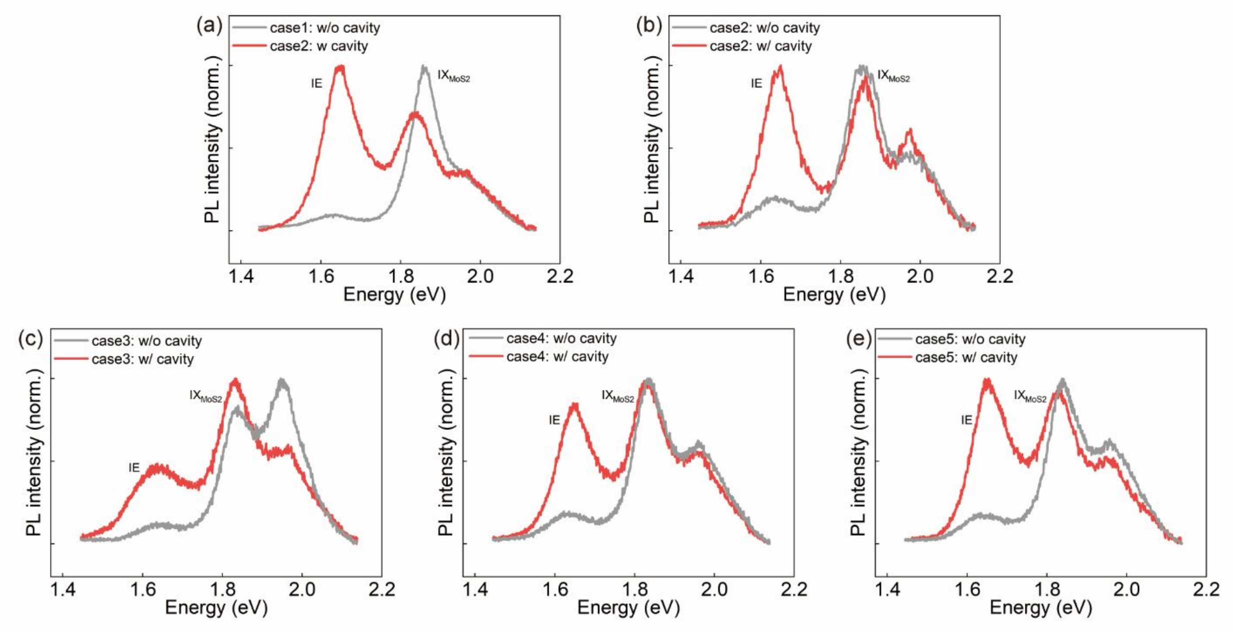

Figure S2. Nanocavity induced-effective modulation of PL spectra of heterostructures observed in many samples.

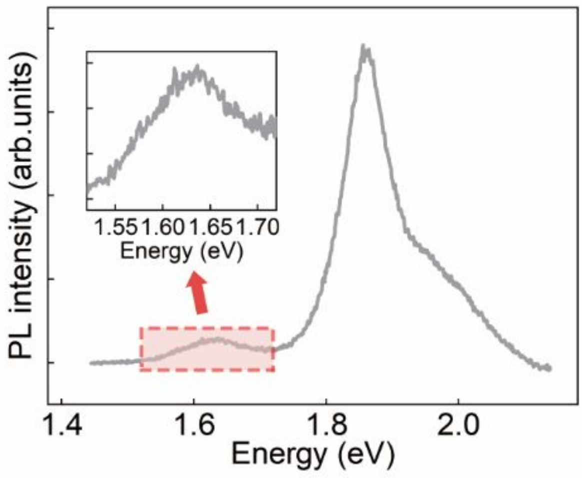

Figure S3. The PL spectrum of the cavity-uncoupled WS_2/MoS_2 heterostructure. The inset shows the magnified view in the spectrum range from 1.52 to 1.72 eV.

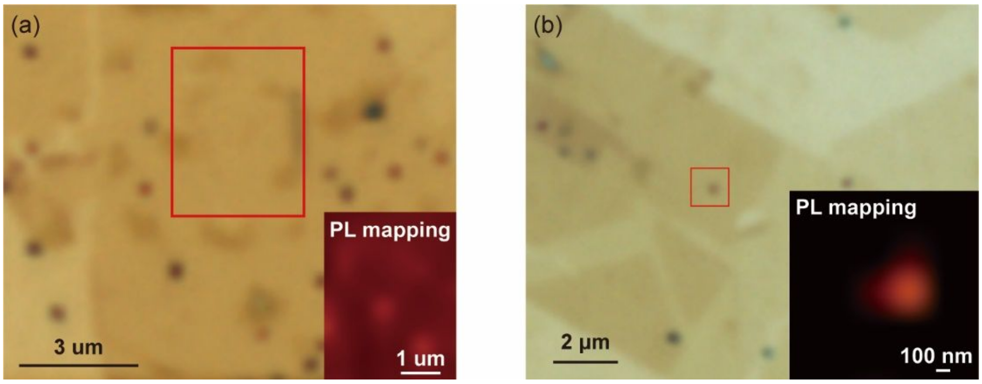

Figure S4. (a) PL mapping of a fabricated heterostructure in the spectral range of interlayer excitons (1.56–1.68 eV) from a region without the nanocavity. The PL intensity shows a generally uniform distribution across an approximately 4.2*3.2 μm^2 zone. Some small spots appeared in the PL mapping may be induced by the speckles formed during the transfer and stacking process of TMD monolayers[26-28]. (b) PL mapping from a region containing a nanocavity. The bright spot indicates the emission intensity of interlayer excitons is selectively enhanced by the nanocavity, while the area outside the nanocavity exhibits uniformly weak PL emission.

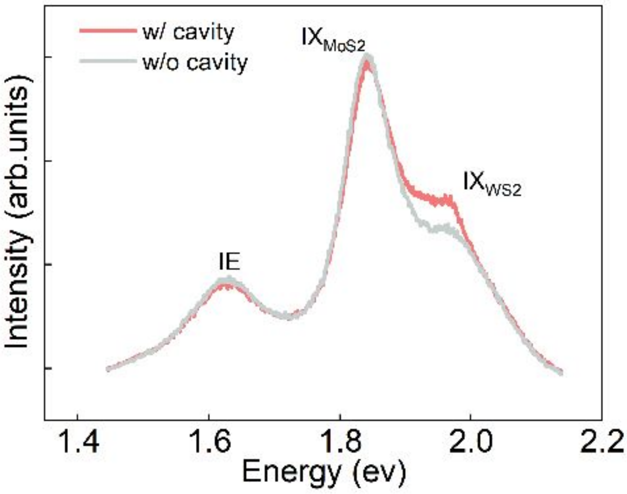

Figure S5. The PL spectrum from the WS_2/MoS_2 heterostructure coupled and uncoupled with a Au-nanocube-on-SiO_2-film cavity.

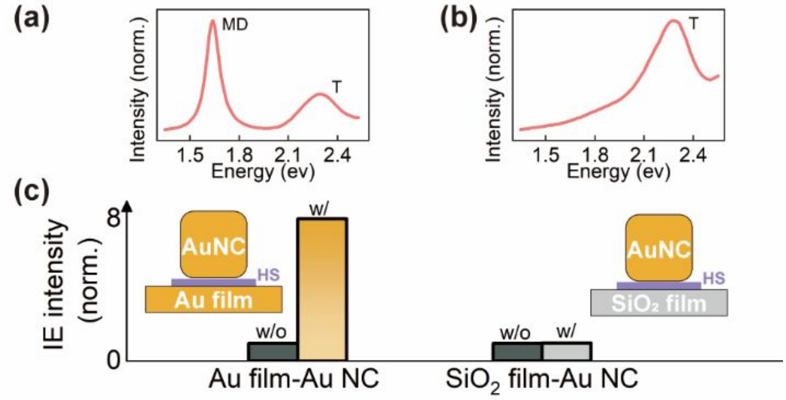

Figure S6. (a, b) The simulated scattering spectrum of the Au-nanocube-on-Au-film cavity, and the Au-nanocube-on-SiO_2-film cavity, respectively. (c) The IE PL enhancement from the heterostructure coupled with the Au-nanocube-on-Au-film cavity and the Au-nanocube-on-SiO_2-film cavity, respectively. Although the overlap of the laser excitation wavelength and the low-energy T mode will induce some near-field excitation enhancement, it can be seen that such an effect alone shows negligible contribution to the IE emission enhancement.

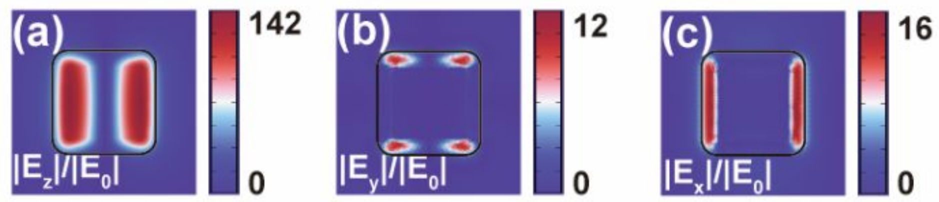

Figure S7. (a–c) The simulated normalized near-field distribution of the electric field components in the middle of the gap between the Au nanocube and the Au film at the MD resonance.

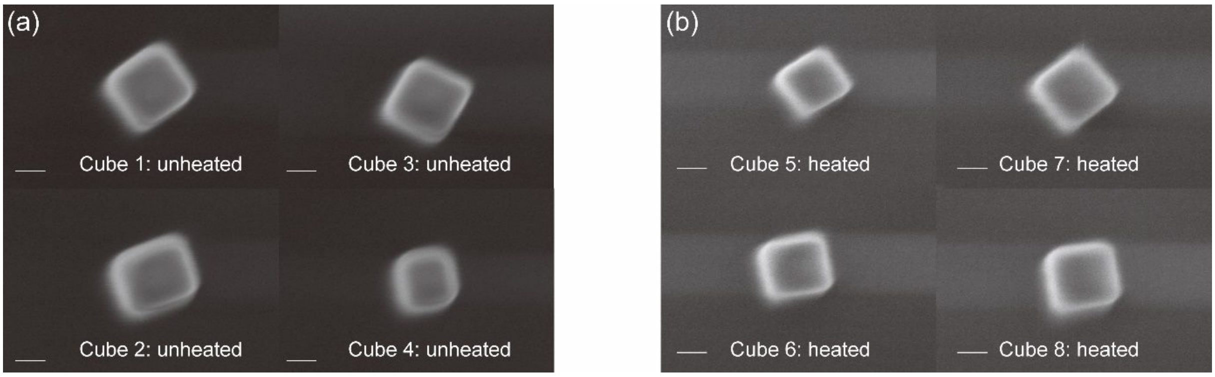

Figure S8. The SEM images of the morphology of Au cubes unheated and heated at 393 K for 3 hours. No significant change in the morphology of Au cubes is observed. Scale bar: 50 nm.

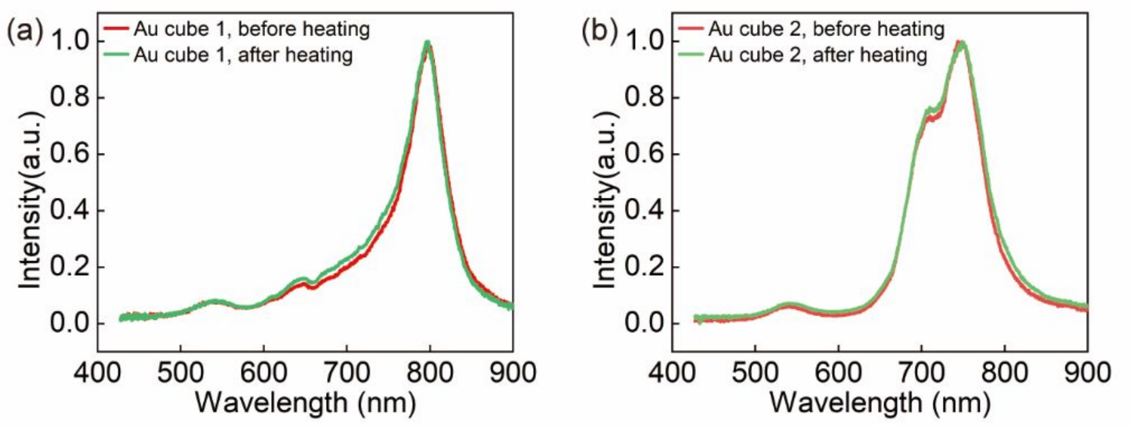

Figure S9. Measured scattering spectra from two NCoM cavities before and after heating at 393 K for 3 hours. There is no significant change on the scattering spectrum of the Au cube after heating.

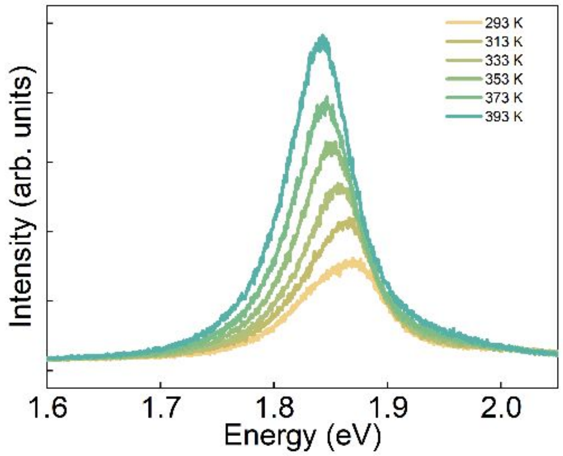

Figure S10. Temperature-dependent PL spectra of the MoS_2 monolayer surrounding the WS_2/MoS_2 heterostructure.

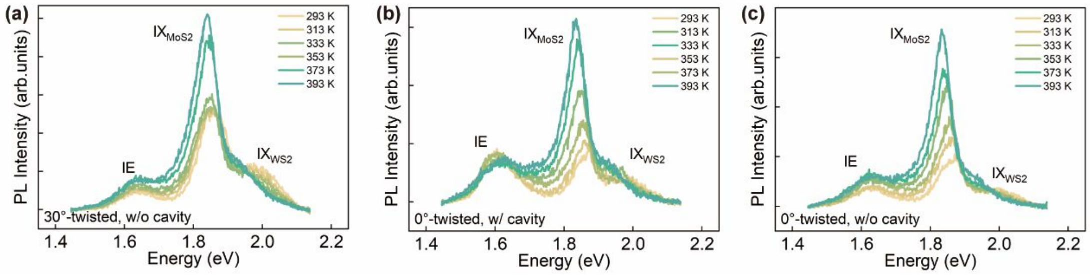

Figure S11. Temperature-dependent PL spectra of the (a) cavity-uncoupled, 30°–twisted heterostructure, (b) cavity-coupled, 0°–twisted heterostructure, (c) cavity-uncoupled, 0°–twisted heterostructure.

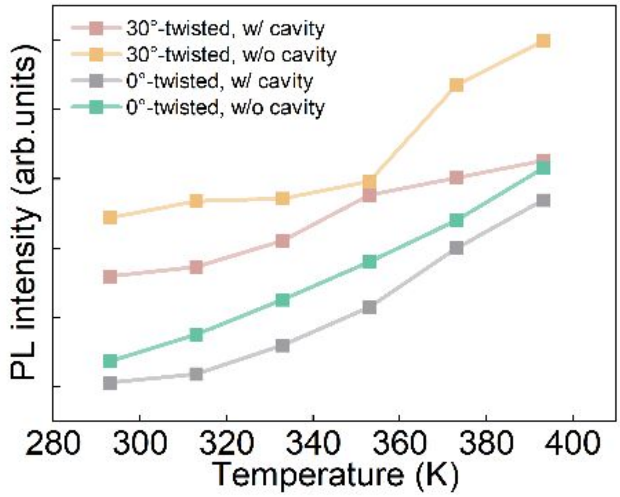

Figure S12. The temperature-dependent PL intensity of MoS_2 from the 0°– and 30°–twisted heterostructures coupled/uncoupled with the nanocavity.

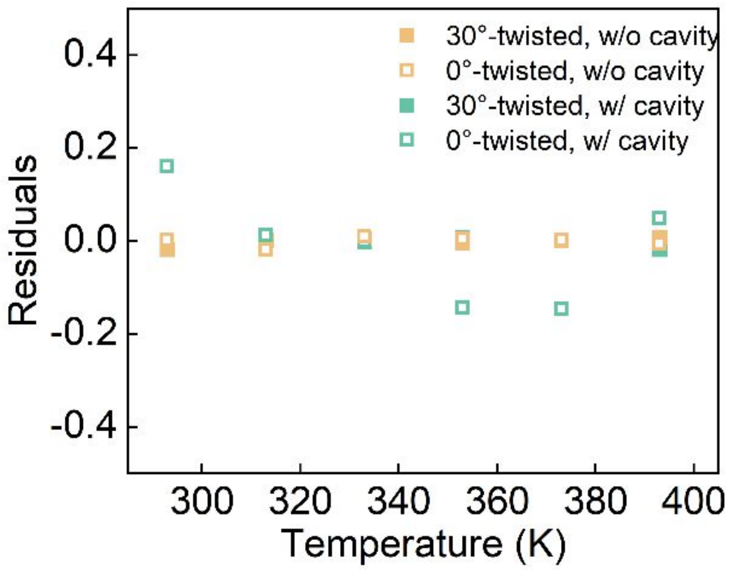

Figure S13. The residual plot of the four fitting cases in Fig. 3c, f.

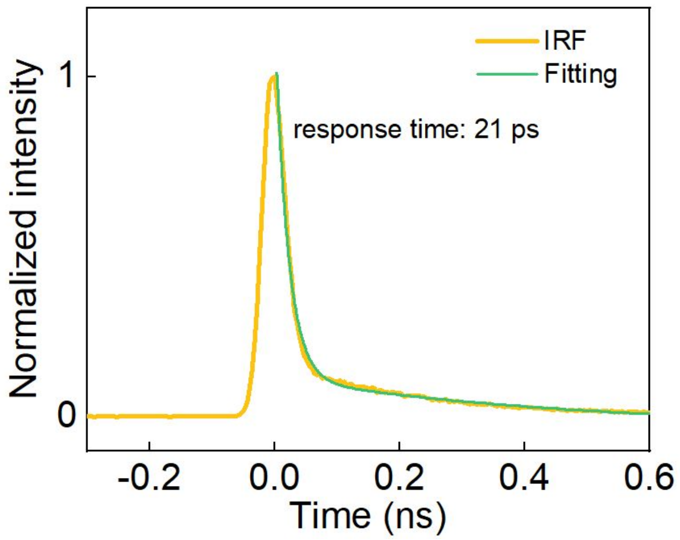

Figure S14. The instrument response function (IRF) of our detection system with a 21 ps response time.

Reference

1. Sun, J.; Hu, H.; Zheng, D.; Zhang, D.; Deng, Q.; Zhang, S.; Xu, H. Light-emitting plexciton: exploiting plasmon–exciton interaction in the intermediate coupling regime. *ACS Nano* **2018,** 12, (10), 10393-10402.
2. Zhang, Y.; Chen, W.; Fu, T.; Sun, J.; Zhang, D.; Li, Y.; Zhang, S.; Xu, H. Simultaneous surface-enhanced resonant Raman and fluorescence spectroscopy of monolayer MoSe2: determination of ultrafast decay rates in nanometer dimension. *Nano Lett.* **2019,** 19, (9), 6284-6291.
3. Tonndorf, P.; Schmidt, R.; Böttger, P.; Zhang, X.; Börner, J.; Liebig, A.; Albrecht, M.; Kloc, C.; Gordan, O.; Zahn, D. R. Photoluminescence emission and Raman response of monolayer MoS 2, MoSe 2, and WSe 2. *Opt. Express* **2013,** 21, (4), 4908-4916.
4. Zhao, W.; Ghorannevis, Z.; Chu, L.; Toh, M.; Kloc, C.; Tan, P.-H.; Eda, G. Evolution of electronic structure in atomically thin sheets of WS2 and WSe2. *ACS Nano* **2013,** 7, (1), 791-797.
5. Sun, J.; Hu, H.; Pan, D.; Zhang, S.; Xu, H. Selectively depopulating valley-polarized excitons in monolayer MoS2 by local chirality in single plasmonic nanocavity. *Nano Lett.* **2020,** 20, (7), 4953-4959.
6. Guo, Y.; Liu, C.; Yin, Q.; Wei, C.; Lin, S.; Hoffman, T. B.; Zhao, Y.; Edgar, J.; Chen, Q.; Lau, S. P. Distinctive in-plane cleavage behaviors of two-dimensional layered materials. *ACS Nano* **2016,** 10, (9), 8980-8988.
7. Choi, J.; Florian, M.; Steinhoff, A.; Erben, D.; Tran, K.; Kim, D. S.; Sun, L.; Quan, J.; Claassen, R.; Majumder, S. Twist angle-dependent interlayer exciton lifetimes in van der Waals heterostructures. *Phys. Rev. Lett.* **2021,** 126, (4), 047401.
8. Mueller, N. S.; Arul, R.; Kang, G.; Saunders, A. P.; Johnson, A. C.; Sánchez-Iglesias, A.; Hu, S.; Jakob, L. A.; Bar-David, J.; de Nijs, B. Photoluminescence upconversion in monolayer WSe2 activated by plasmonic cavities through resonant excitation of dark excitons. *Nat. Commun.* **2023,** 14, (1), 5726.
9. Penzo, E.; Loiudice, A.; Barnard, E. S.; Borys, N. J.; Jurow, M. J.; Lorenzon, M.; Rajzbaum, I.; Wong, E. K.; Liu, Y.; Schwartzberg, A. M. Long-range exciton diffusion in two-dimensional assemblies of cesium lead bromide perovskite nanocrystals. *ACS Nano* **2020,** 14, (6), 6999-7007.
10. Johnson, P. B.; Christy, R.-W. Optical constants of the noble metals. *Phys. Rev. B* **1972,** 6, (12), 4370.
11. Waxenegger, J.; Trügler, A.; Hohenester, U. Plasmonics simulations with the MNPBEM toolbox: Consideration of substrates and layer structures. *Comput. Phys. Commun.* **2015,** 193, 138-150.
12. Li, Y.; Liu, W.; Xu, H.; Zhang, C.; Yang, L.; Yue, W.; Liu, Y. Abnormal high-temperature luminescence enhancement observed in monolayer MoS 2 flakes: thermo-driven transition from negatively charged trions to neutral excitons. *J. Mater. Chem. C* **2016,** 4, (39), 9187-9196.
13. Roy, S.; Sharbirin, A. S.; Lee, Y.; Kim, W. B.; Kim, T. S.; Cho, K.; Kang, K.; Jung, H. S.; Kim, J. Measurement of quantum yields of monolayer TMDs using dye-dispersed

PMMA thin films. *Nanomaterials* **2020,** 10, (6), 1032.
14. Akselrod, G. M.; Ming, T.; Argyropoulos, C.; Hoang, T. B.; Lin, Y.; Ling, X.; Smith, D. R.; Kong, J.; Mikkelsen, M. H. Leveraging nanocavity harmonics for control of optical processes in 2D semiconductors. *Nano Lett.* **2015,** 15, (5), 3578-3584.
15. Mouri, S.; Miyauchi, Y.; Matsuda, K. Tunable photoluminescence of monolayer MoS2 via chemical doping. *Nano Lett.* **2013,** 13, (12), 5944-5948.
16. Hoang, T.; Akselrod, G.; Argyropoulos, C.; Huang, J.; Smith, D.; Mikkelsen, M., Ultrafast spontaneous emission source using plasmonic nanoantennas Nat. Commun: 2015.
17. Rose, A.; Hoang, T. B.; McGuire, F.; Mock, J. J.; Ciracì, C.; Smith, D. R.; Mikkelsen, M. H. Control of radiative processes using tunable plasmonic nanopatch antennas. *Nano Lett.* **2014,** 14, (8), 4797-4802.
18. Russell, K. J.; Liu, T.-L.; Cui, S.; Hu, E. L. Large spontaneous emission enhancement in plasmonic nanocavities. *Nat. Photonics* **2012,** 6, (7), 459-462.
19. Heo, H.; Sung, J. H.; Cha, S.; Jang, B.-G.; Kim, J.-Y.; Jin, G.; Lee, D.; Ahn, J.-H.; Lee, M.-J.; Shim, J. H. Interlayer orientation-dependent light absorption and emission in monolayer semiconductor stacks. *Nat. Commun.* **2015,** 6, (1), 7372.
20. Nayak, P. K.; Horbatenko, Y.; Ahn, S.; Kim, G.; Lee, J.-U.; Ma, K. Y.; Jang, A.-R.; Lim, H.; Kim, D.; Ryu, S. Probing evolution of twist-angle-dependent interlayer excitons in MoSe2/WSe2 van der Waals heterostructures. *ACS Nano* **2017,** 11, (4), 4041-4050.
21. Shi, J.; Li, Y.; Zhang, Z.; Feng, W.; Wang, Q.; Ren, S.; Zhang, J.; Du, W.; Wu, X.; Sui, X. Twisted-angle-dependent optical behaviors of intralayer excitons and trions in WS2/WSe2 heterostructure. *ACS Photonics* **2019,** 6, (12), 3082-3091.
22. Tebyetekerwa, M.; Zhang, J.; Saji, S. E.; Wibowo, A. A.; Rahman, S.; Truong, T. N.; Lu, Y.; Yin, Z.; Macdonald, D.; Nguyen, H. T. Twist-driven wide freedom of indirect interlayer exciton emission in MoS2/WS2 heterobilayers. *Cell Rep. Phys. Sci.* **2021,** 2, (8).
23. Ceballos, F.; Bellus, M. Z.; Chiu, H.-Y.; Zhao, H. Ultrafast charge separation and indirect exciton formation in a MoS2–MoSe2 van der Waals heterostructure. *ACS Nano* **2014,** 8, (12), 12717-12724.
24. Hong, X.; Kim, J.; Shi, S.-F.; Zhang, Y.; Jin, C.; Sun, Y.; Tongay, S.; Wu, J.; Zhang, Y.; Wang, F. Ultrafast charge transfer in atomically thin MoS2/WS2 heterostructures. *Nat. Nanotechnol.* **2014,** 9, (9), 682-686.
25. Zhu, H.; Wang, J.; Gong, Z.; Kim, Y. D.; Hone, J.; Zhu, X.-Y. Interfacial charge transfer circumventing momentum mismatch at two-dimensional van der Waals heterojunctions. *Nano Lett.* **2017,** 17, (6), 3591-3598.
26. Jain, A.; Bharadwaj, P.; Heeg, S.; Parzefall, M.; Taniguchi, T.; Watanabe, K.; Novotny, L. Minimizing residues and strain in 2D materials transferred from PDMS. *Nanotechnology* **2018,** 29, (26), 265203.
27. Pizzocchero, F.; Gammelgaard, L.; Jessen, B. S.; Caridad, J. M.; Wang, L.; Hone, J.; Bøggild, P.; Booth, T. J. The hot pick-up technique for batch assembly of van der Waals heterostructures. *Nat. Commun.* **2016,** 7, (1), 11894.
28. Purdie, D. G.; Pugno, N.; Taniguchi, T.; Watanabe, K.; Ferrari, A.; Lombardo, A.

Cleaning interfaces in layered materials heterostructures. *Nat. Commun.* **2018,** 9, (1), 5387.
